# Release Kinetics Model Fitting of Drugs with Different Structures from Viscose Fabric

**DOI:** 10.3390/ma16083282

**Published:** 2023-04-21

**Authors:** Weiwei Zhu, Jiajie Long, Meiwu Shi

**Affiliations:** College of Textile and Clothing Engineering, Suzhou University, Suzhou 215127, China; wwzhu1@suda.edu.cn

**Keywords:** release kinetics model, drug-loaded viscose fabric, supercritical CO_2_ fluid, Fickian diffusion

## Abstract

(1) Background: It is simpler and more environmentally friendly to use supercritical CO_2_ fluid technology to process skincare viscose fabrics. Therefore, it is significant to study the release properties of drug-loaded viscose fabrics to choose suitable skincare drugs. In this work, the release kinetics model fittings were investigated in order to clarify the release mechanism and provide a theoretical basis for processing skincare viscose fabrics with supercritical CO_2_ fluid. (2) Methods: Nine kinds of drugs with different substituent groups, different molecular weights, and different substitution positions were loaded onto viscose fabrics using supercritical CO_2_ fluid. Then, the drug-loaded viscose fabrics were placed in an ethanol medium, and the release curves were drawn. Finally, the release kinetics were fitted using zero-order release kinetics, the first-order kinetics model, the Higuchi model, and the Korsmeyer–Peppas model. (3) Results: The Korsmeyer–Peppas model was the best-fitting model for all the drugs. Drugs with different substituent groups were released via a non-Fickian diffusion mechanism. On the contrary, other drugs were released via a Fickian diffusion mechanism. (4) Conclusions: In view of the release kinetics, it was found that the viscose fabric can swell when a drug with a higher solubility parameter is loaded onto it using supercritical CO_2_ fluid, and the release rate is also slower.

## 1. Introduction

Viscose fiber is the main variety of regenerated fiber, and its raw material comes from natural cellulose with the largest reserves and the most sustainable development on Earth [1]. Viscose fiber has been widely used in underwear products because of its moisture absorption, air permeability, antistatic, good color fastness, and spinning properties. However, with the development of society, people have a tendency to choose functional fabrics, such as antimicrobial [2], personal cooling [3], or waterproof [4] ones. Therefore, if viscose fabric is endowed with skincare functionality such as antioxidant, skin whitening, moisturizing, etc., its additional value and consumer market will improve significantly in the field of underwear.

Some methods have been utilized to fabricate skincare textiles including blended spinning technology [5] and microencapsulation [6]. A large quantity of drugs (2–10 wt%) can be mixed into the fiber through blended spinning, but it is not suitable for thermosensitive drugs [7]. In addition, it leads to wastage of the drug because most of the drug is distributed in the interior of the fiber and cannot be released, and the properties of the fiber can be affected by the drugs. Microencapsulation can not only protect drugs from the environmental impact but also slow down the release rate of drugs such as vitamins [8] and essential oils [9]. However, the preparation process is complex and requires the usage of organic solvents [10,11]. When the microcapsule is loaded on the fabric, it can easily affect the touch feeling because of the joint of the cross-linking agent.

By contrast, supercritical CO_2_ fluid technology is a simpler, green, and efficient finishing technology that can dissolve or carry drugs into the surface and interior of the fabric, and the drugs can be released in a totally controlled way [12,13]. After depressurization, the fluid directly transforms into CO_2_ gas and is recycled, so that a functional fabric loaded with drugs is obtained [14,15]. The whole process is free of any solvent residue and operates under a mild temperature, which is advantageous for processing thermosensitive drugs [16,17]. Thus, it has more strengths to offer for the processing of skincare viscose fabric compared to the existing technology.

Supercritical CO_2_ fluid technology is, therefore, more suitable for fabricating skincare viscose fabrics, but relevant research is scarce. Furthermore, drug loading capacity and release property are important evaluation indexes for fabricating a drug-loaded substrate, such as acetazolamide- and timolol-maleate-loaded lenses [18] or thymol-loaded cellulose acetate [12]. Therefore, it is significant to study the loading capacity and release property of drug-loaded viscose fabrics processed using supercritical CO_2_ fluid technology. This has been researched in a previous work [19]: Nine kinds of drugs with different substituent groups, different molecular weights, and different substitution positions were loaded onto a viscose fabric using supercritical CO_2_ fluid in order to investigate the effect of the drugs with different structures on the solubility, drug-loaded capacity, and release property. It was found that differences in the structures of the drugs obviously resulted in the variation of the drug loading capacity and release property. However, the variation of the release curves for the nine drugs over time, which has a significant effect on the functionality of the skincare viscose fabric, was not analyzed in depth. Therefore, a more in-depth investigation to clarify the release mechanism is necessary.

In this work, the release curves of the nine drugs from the viscose fabrics were fitted using zero-order release kinetics ($Q_{t}=K_{0}\cdot t+b_{0}$), the first-order kinetics model ($Q_{t}=K_{1}\cdot e^{at}+b_{1}$), the Higuchi model ($Q_{t}=K_{H}\cdot t^{1/2}+b_{H}$), and the Korsmeyer–Peppas model ($Q_{t}=K\cdot t^{n}$), which are usually used to describe drug release from polymeric systems and to establish the predictability of the temporal drug release [20,21,22,23]. The aim was to choose the most suitable model and clarify the effect of variation of the structures of the drugs on the relevant parameters, as well as the release mechanism of drug-loaded viscose fabric processed using supercritical CO_2_ fluid. The model fitting can be an effective method to deduce the release process and provide a theoretical basis for choosing suitable drugs to fabricate skincare viscose fabrics with supercritical CO_2_ fluid technology.

## 2. Materials and Methods

### 2.1. Materials

**Figure 1:**
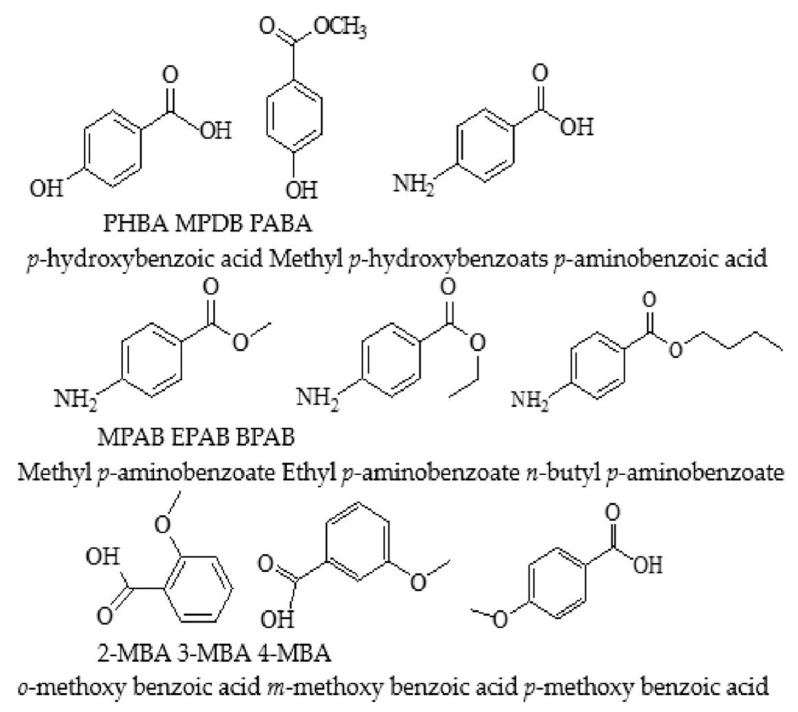
The structure of nine kinds of drugs [19].

The nine drugs have been introduced in the previous paper [19] and are described in Figure 1. They were, respectively, p-hydroxybenzoic acid (PHBA), methyl p-hydroxybenzoate (MPDB), p-aminobenzoic acid (PABA), methyl p-aminobenzoate (MPAB), ethyl p-aminobenzoate (EPAB), n-butyl p-aminobenzoate (BPAB), o-methoxy benzoic acid (2-MBA), m-methoxy benzoic acid (3-MBA), and p-methoxy benzoic acid (4-MBA), which were purchased in analytical reagent grade from J&K Co. Ltd. (Beijing, China). The viscose fabric was pretreated and provided by Shandong Woyuan Newtype Fabrics Co. Ltd. (Shandong, China). Pure carbon dioxide gas (purity ≥99.8 vol.%) was purchased from Chengxing Industrial Gas Supply Co. Ltd. (Suzhou, China). Ethanol was supplied by the Sinopharm Group and used to dissolve the employed drugs.

### 2.2. Methods

#### 2.2.1. Impregnation of Drugs into Viscose Fabric Using Supercritical CO_2_ Fluid

The supercritical CO_2_ fluid equipment diagram has been depicted previously [19]. The viscose fabric was wrapped around a cylindrical shelf and placed into the autoclave. The drug with a 5% dosage of the mass of the viscose fabric was added in the bottom of the autoclave. They were separated from each other. Then, the autoclave was closed, and the condensational CO_2_ liquid was introduced, which transformed into supercritical CO_2_ fluid with increasing temperature (55 °C) and increasing pressure (20 MPa). The circulating pump was opened, and the fluid was circulated at a ratio of 1 min:10 min until the experiment was over at 120 min. Finally, the pressure was released, the CO_2_ gas was recycled, and the drug-loaded viscose fabric was taken out.

#### 2.2.2. The Quantitative Evaluation of Release Amount of Drug-Loaded Viscose Fabric

The drug-loaded viscose fabric was extracted with ethanol and the Soxhlet method at 105 °C for 6 h. All the utilized drugs had good solubility in ethanol, which could avoid the effect of a solvent on the release property of different drugs from the viscose fabric. Then, the ethanol containing the extracted drug was transferred into a 250.0 mL volumetric flask, and the extracted viscose fabric was dried at 105 °C for 4 h. A calibration curve for quantitative evaluation of the drug loading capacity for each drug was drawn using an ultraviolet visible spectrophotometer (TU-1810, Beijing Purkinje General Instrument Co., Ltd., Beijing, China) at wavelengths from 190.0 nm to 400.0 nm [19]. The calculation of the drug loading capacity onto the viscose fabric is shown in Equation (1):

(1) $$LA=\frac{(k\cdot A+b)\times V}{G}$$

where *LA* refers to the drug loading capacity of the each extracted drug against the viscose fabric after being extracted and drying (unit g∙g^−1^). *k*, *b* are, respectively, the slope value and the constant value of the calibration curve. *A* is the characteristic absorbance of the drug dissolved in ethanol. *V* is the constant volume of ethanol with dissolving drug, 250 mL. *G* is the mass of the extracted viscose fabric after drying.

Meanwhile, two pieces of drug-loaded viscose fabrics with dimensions of 4.5 cm × 4.5 cm were utilized and immersed into ethanol as a solvent with a volume of 150.0 mL at a room temperature of 25.0 °C, which was used for constructing the release curve. A small amount of solution was taken out for spectrophotometric measurement at a certain time interval. When the measurement was finished, the solution was returned. After the process was over, the viscose fabric was taken out for weighing after drying at 105 °C for 4 h. The cumulative release percentage of each drug from the viscose fabric was calculated as in Equation (2):

(2) $$Q_{t}=\frac{\frac{(k\cdot A_{t}+b)\times V_{1}}{G_{1}}}{LA}\times 100\%$$

where *Q_t_* is the releasing capacity of drugs from the viscose fabric against the total drug-loaded capacity in a unit of (g∙g^−1^) at t time. *A_t_* is the characteristic absorbance of the released drug in ethanol at t time. *V*_1_ is the volume of ethanol, 150 mL. *G*_1_ is the mass of the two pieces of viscose fabric after drying.

## 3. Results

### 3.1. Release Kinetics Model Fitting of Drugs with Different Substituent Groups from Viscose Fabric

**Figure 2:**
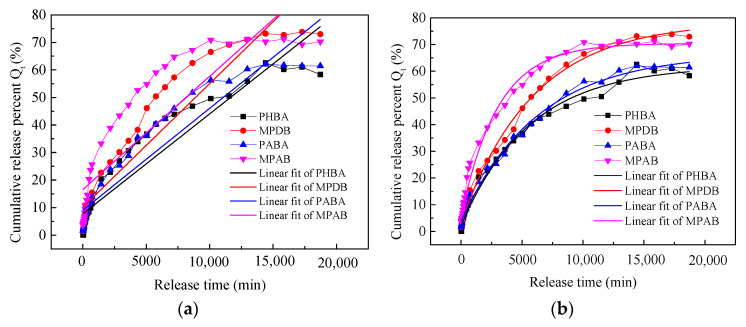

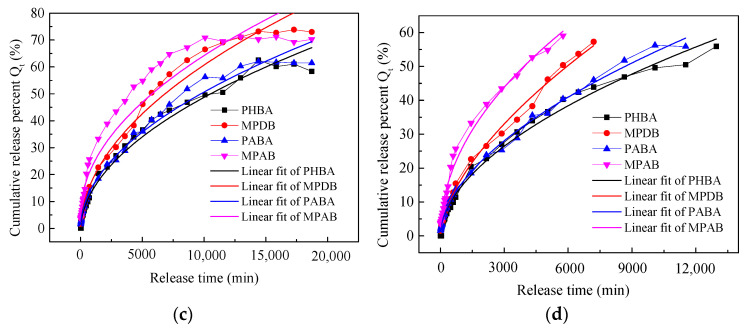
The release kinetics model fitting curves of PHBA, MPDB, PABA, and MPAB drugs: (**a**) zero-order release kinetics; (**b**) first-order kinetics model; (**c**) Higuchi model; and (**d**) Korsmeyer–Peppas model.

The drugs PHBA, MPDB, PABA, and MPAB have different polar groups, which are, respectively, hydroxyl groups (-OH), carboxy groups (-COOH), and amidogen groups (-NH_2_) for the last two. The release curves depicted in the previous work were fitted using zero-order release kinetics, the first-order kinetics model, the Higuchi model, and the Korsmeyer–Peppas model. The fitted curves and the relevant equations are described in Figure 2 and Table 1.

The linear fit curves and their corresponding equations are shown in Figure 2 and Table 1. Adj. R-Square, Pearson’s r, Residual Sum of Squares, and Reduced Chi-Sqr were used to test the applicability of the release kinetics models [24]. Adj. R-Square is the most common parameter used to choose the most suitable model [25]. A higher Adj. R-Square and Pearson’s r represent a model that is more applicable to the release curve. On the contrary, a smaller Residual Sum of Squares and Reduced Chi-Sqr represent a better kinetic model [24]. It was found that the first-order kinetics model and the Korsmeyer–Peppas model were well in accordance with the drug release pattern as shown in Figure 2 and Table 1. The values of Adj. R-Square were above 0.99. Furthermore, the Residual Sum of Square and Reduced Chi-Sqr were smaller than those of the other two models as described in Table 1. Compared to the first-order kinetics model, the Korsmeyer–Peppas model was the best fitting model because of its smaller Residual Sum of Squares and Reduced Chi-Sqr.

The first-order release model represents that the drugs are mainly adsorbed to the inner wall of the substrate [26]. Therefore, it indicates that PHBA, MPDB, PABA, and MPAB may be mainly distributed in the interior of the viscose fabric processed using supercritical CO_2_ fluid. This equation of the Korsmeyer–Peppas model is valid for the first 60% of the fractional release, which is useful to describe various mechanisms of transport including Fickian diffusion and non-Fickian transport [20,27]. K is the release rate constant, and n is the release exponent for the Korsmeyer–Peppas model. The value of “*n*” predicts the release mechanism of the drug. When *n* is <0.45, it means that the release pattern belongs to the Fickian diffusion mechanism, 0.45 < *n* < 0.89 to non-Fickian transport, *n* = 0.89 to Case II transport, and *n* > 0.89 to super Case II transport in the case of a cylinder [24,28]. Viscose fabric is made up of viscose fibers, which can be regarded as thin cylinders. In Table 1, the n value of the Korsmeyer–Peppas models for the release of the PHBA, MPDB, PABA, and MPAB drugs from the viscose fabric is between 0.45 and 0.89, which suggests that their release property follows non-Fickian transport. In this case, it indicates that Fickian diffusion is the predominant mechanism. However, the matrix swelling reaches equilibrium much faster than the drug release rate, so that a major part of the diffusion process takes place on the already swollen polymeric matrix [29]. It suggests that the viscose fabric was swollen when the PHBA, MPDB, PABA, and MPAB drugs were impregnated into it by the supercritical CO_2_ fluid. As a result, the release pattern of the PHBA, MPDB, PABA, and MPAB drugs from the viscose fabric in ethanol medium deviated from Fickian diffusion to a degree.

### 3.2. Release Kinetics Model Fitting of Drugs with Different Molecular Weights from Viscose Fabric

**Figure 3:**
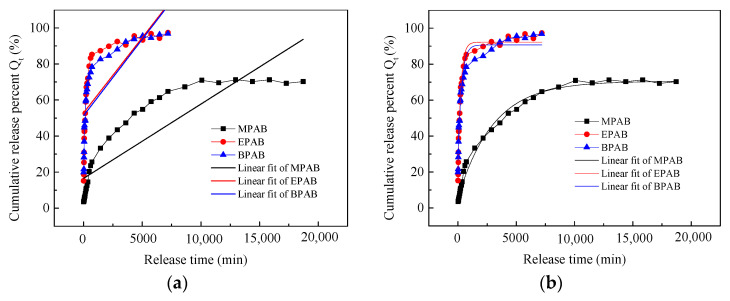

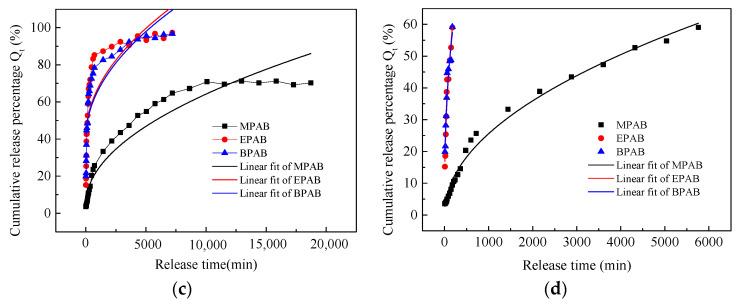
The release kinetics model fitting curves of MPAB, EPAB, and BPAB drugs: (**a**) zero-order release kinetics; (**b**) first-order kinetics model; (**c**) Higuchi model; and (**d**) Korsmeyer–Peppas model.

The drugs MPAB, EPAB, and BPAB have different molecular weights and differ in the number of methylene groups (-CH_2_-). EPAB has one more methylene group than MPAB, and BPAB has three more methylene groups than MPAB as shown in Figure 1. The release curves were also fitted using zero-order release kinetics, the first-order kinetics model, the Higuchi model, and the Korsmeyer–Peppas model. The fitted curves and the relevant equations are described in Figure 3 and Table 2.

The linear fit curves and their corresponding equations are shown in Figure 3 and Table 2. As described in Table 2, the Adj. R-Square values of the first-order kinetics model and the Korsmeyer–Peppas model for the MPAB, EPAB, and BPAB drugs were much higher than those for zero-order release kinetics and the Higuchi model. Furthermore, the Residual Sum of Squares and Reduced Chi-Sqr of the first-order kinetics model and the Korsmeyer–Peppas model were much lower. Therefore, the first-order kinetics model and the Korsmeyer–Peppas model were the better-fitting models for the release curves of the MPAB, EPAB, and BPAB drugs from viscose fabrics based on supercritical CO_2_ fluid technology. Although the Adj. R-Square of the Korsmeyer–Peppas model was a little lower than that of the first-order kinetics model, the Korsmeyer–Peppas model was more suitable for fitting the releasing curve of the MPAB, EPAB, and BPAB drugs from viscose fabrics because of its much lower Residual Sum of Squares and Reduced Chi-Sqr. In addition, the n value of the Korsmeyer–Peppas model was above 0.45 for the MPAB drug, which was analyzed in Section 3.1, but it was less than 0.45 for the EPAB and BPAB drugs. Meanwhile, the k values of the Korsmeyer–Peppas model for the EPAB and BPAB drugs were, respectively, 6.2303 and 8.2661, which are much higher than that of MPAB.

From the above, it can be concluded that the release of the EPAB and BPAB drugs from viscose fabrics followed a Fickian diffusion mechanism because the n values were lower than 0.45. Conversely, the release pattern of the MPAB drug followed a non-Fickian diffusion mechanism as described in Section 3.1. This suggests that the increase in the number of methylene groups resulted in the transformation of the release property of the drug from viscose fabrics manufactured using supercritical CO_2_ fluid from non-Fickian diffusion to Fickian diffusion. Moreover, a higher k value may suggest that there exists a burst release phenomenon of the drug from the polymeric matrix [30]. In fact, in Figure 2, the release rate is really very quick at the first stage for the EPAB and BPAB drugs, and the corresponding k values are much higher. It suggests that the k values can represent the burst effect of the EPAB and BPAB drugs well. The reasons for the above phenomena maybe the different distribution of the MPAB, EPAB, and BPAB drugs onto the viscose fabric. EPAB and BPAB drugs with higher molecular weights do not easily enter into the interior of the viscose fabric or swell the viscose fabric through the supercritical CO_2_ fluid. After depressurization, most of the EPAB and BPAB drugs were distributed on the surface of viscose fiber; therefore, the EPAB and BPAB drugs were released into the ethanol medium at a quicker rate via a Fickian diffusion mechanism. On the contrary, MPDB was carried into the interior of the viscose fiber by the supercritical CO_2_ fluid and had a swelling effect. As a consequence, the MPDB drug was released into the ethanol medium at a relative slow rate via a non-Fickian diffusion mechanism.

### 3.3. Release Kinetics Model Fitting of Drugs with Different Substitution Positions from Viscose Fabric

**Figure 4:**
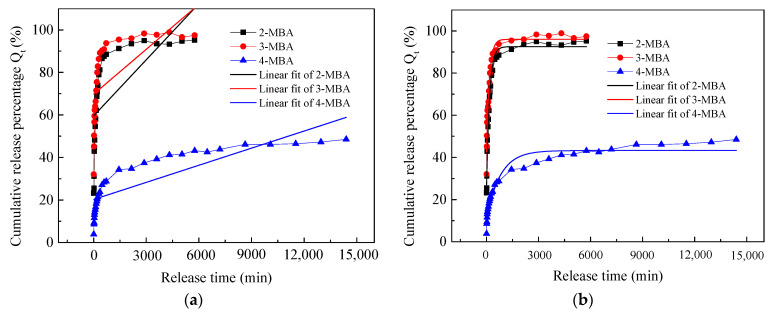

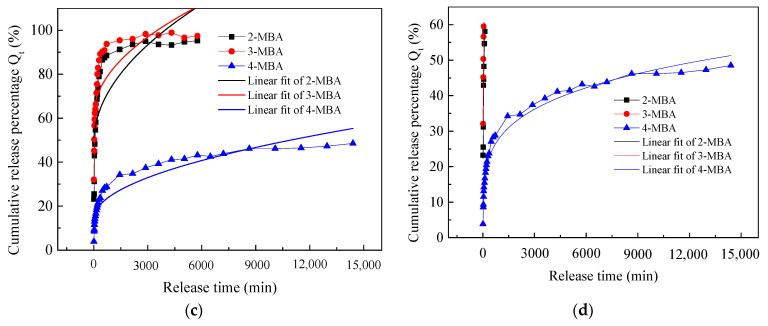
The release kinetics model fitting curves of 2-MBA, 3-MBA, and 4-MBA drugs: (**a**) zero-order release kinetics; (**b**) first-order kinetics model; (**c**) Higuchi model; and (**d**) Korsmeyer–Peppas model.

The drugs 2-MBA, 3-MBA, and 4-MBA have different substitution positions with the carboxyl group, methoxy group, and carboxyl groups, respectively, located in the ortho, meta, para position of the methoxy group. The release curves were fitted using zero-order release kinetics, the first-order kinetics model, the Higuchi model, and the Korsmeyer–Peppas model. The fitted curves and the relevant equations are described in Figure 4 and Table 3.

The linear fit curves and their corresponding equations are shown in Figure 4 and Table 3. Similarly, the fitting curves of the first-order kinetics model and the Korsmeyer–Peppas model were more in accordance with the release curve of the 2-MBA, 3-MBA, and 4-MBA drugs from the viscose fabric as shown in Figure 4b,d. Furthermore, the Adj. R-Square values of first-order kinetics model and Korsmeyer–Peppas model were also much higher than those of zero-order release kinetics and the Higuchi model, and the corresponding Residual Sum of Squares and Reduced Chi-Sqr were much lower, as in Table 3. Therefore, the first-order kinetics model and the Korsmeyer–Peppas model were more suitable for describing the releasing of the 2-MBA, 3-MBA, and 4-MBA drugs from the viscose fabric. Compared to the first-order kinetics model, the Residual Sum of Squares and Reduced Chi-Sqr of the Korsmeyer–Peppas model were much lower except that the Reduced Chi-Sqr was a little higher for the 2-MBA drug; therefore, the Korsmeyer–Peppas model was the best-fitted model. The n values of the Korsmeyer–Peppas model for the 2-MBA, 3-MBA, and 4-MBA drugs were lower than 0.45, which suggests that the release of the 2-MBA, 3-MBA, and 4-MBA drugs from the viscose fabric followed a Fickian diffusion mechanism.

From the above, it can be seen that the release property of all the nine drugs can be fitted by the Korsmeyer–Peppas model well. However, the release of the PHBA, MPDB, PABA, and MPAB drugs from the viscose fabric followed a non-Fickian diffusion mechanism, and the release of the EPAB, BPAB, 2-MBA, 3-MBA, and 4-MBA drugs from the viscose fabric followed a Fickian diffusion mechanism based on the value of “n”. Moroever, the corresponding k values of the Korsmeyer–Peppas models for the PHBA, MPDB, PABA, and MPAB drugs (k < 0.87) were much lower than those for the EPAB, BPAB, 2-MBA, 3-MBA, and 4-MBA drugs (k > 6.09), which suggests that there exists a burst effect for the EPAB, BPAB, 2-MBA, 3-MBA, and 4-MBA drugs. A good interaction between drug and substrate can contribute to the swelling effect of supercritical CO_2_ fluid on the substrate and slow the release rate of drugs from the substrate [31]. It is known that the molecular chains of viscose fibers have a large amount of hydroxyl groups, which are the polar groups. The solubility parameters for PHBA, MPDB, PABA, MPAB, EPAB, BPAB, 2-MBA, 3-MBA, and 4-MBA drugs were, respectively, 27.281 (MJ/m^3^)^1/2^, 25.587 (MJ/m^3^)^1/2^, 24.340 (MJ/m^3^)^1/2^, 21.574 (MJ/m^3^)^1/2^, 21.006 (MJ/m^3^)^1/2^, 20.161 (MJ/m^3^)^1/2^, 20.056 (MJ/m^3^)^1/2^, 20.056 (MJ/m^3^)^1/2^, and 20.056 (MJ/m^3^)^1/2^ calculated by the method of Hoftyzer–Van Krevelen [32,33,34], which indicate a higher polarity for PHBA, MPDB, PABA, and MPAB because of their higher solubility parameters. It is concluded that the interaction of polar groups between drugs and the viscose fabric has an effect on the diffusion mechanism and the burst effect. A better interaction between the PHBA, MPDB, PABA, and MPAB drugs and the viscose fiber resulted in the swelling effect of supercritical CO_2_ fluid on viscose fabric and slowed the release rate. Finally, the release property of the PHBA, MPDB, PABA, and MPAB drugs followed non-Fickian diffusion and the corresponding release rate was slower. On the contrary, a worse interaction between the EPAB, BPAB, 2-MBA, 3-MBA, and 4-MBA drugs and the viscose fiber resulted in the drugs being released via Fickian diffusion, and burst effects occurred.

## 4. Conclusions

The release property of the PHBA, MPDB, PABA, MPAB, EPAB, BPAB, 2-MBA, 3-MBA, and 4-MBA drugs with different polar groups, different molecular weights, and different substitution positions from drug-loaded viscose fabric impregnated by supercritical CO_2_ fluid were fitted using zero-order release kinetics, the first-order kinetics model, the Higuchi model, and the Korsmeyer–Peppas model. It was found that the Korsmeyer–Peppas model was the best-fitting model for all the drugs. Moreover, the difference in the release mechanism for drugs with different substituent groups, different molecular weights, and different substitution positions can be represented by the solubility parameter. The n value of the kinetic model equations was higher than 0.45 and the corresponding k was lower than 0.87 for PHBA, MPDB, PABA, and MPAB drugs having a higher solubility parameter, which means that the drugs were released via a non-Fickian diffusion mechanism. It suggests that a better interaction between the PHBA, MPDB, PABA, and MPAB drugs and the viscose fabric resulted in the supercritical CO_2_ fluid having a swelling effect on the viscose fiber and slowed the release rate of drugs from the viscose fabric. On the contrary, the n value was lower than 0.45 and k was higher than 6.09 for the EPAB, BPAB, 2-MBA, 3-MBA, and 4-MBA drugs having a lower solubility parameter, which means that the drugs were released via a Fickian diffusion mechanism. It indicates that a worse interaction between the EPAB, BPAB, 2-MBA, 3-MBA, and 4-MBA drugs and the viscose fiber resulted in the drugs being released quickly from the viscose fabric.

## Figures and Tables

**Table 1:** The release kinetics model fitting parameters of PHBA, MPDB, PABA, and MPAB drugs.

Drugs	$\mathrm{Zero}-\mathrm{Order}\;\mathrm{Release}\;\mathrm{Kinetics}\;(Q_{t}=K_{0}\cdot t+b_{0})$			
	Equation	Adj. R-Square	Residual Sum of Squares	Pearson’s r
PHBA	$Q_{t}=0.00363\cdot t+7.7436$	0.88534	1824.73559	0.94277
MPDB	$Q_{t}=0.00449\cdot t+10.15534$	0.8879	2720.47914	0.94409
PABA	$Q_{t}=0.00371\cdot t+9.04042$	0.89637	1695.53215	0.94843
MPAB	$Q_{t}=0.00412\cdot t+16.55115$	0.76907	5412.90171	0.88095
	First-order kinetics model ($Q_{t}=K_{1}\cdot e^{at}+b_{1}$)			
	Equation	Adj. R-Square	Residual Sum of Squares	Reduced Chi-Sqr
PHBA	$Q_{t}=61.99435-59.78837\cdot e^{-0.00017754\cdot t}$	0.99108	137.58277	4.43815
MPDB	$Q_{t}=78.5777-75.07381\cdot e^{-0.0001685\cdot t}$	0.99587	96.99985	3.12903
PABA	$Q_{t}=66.54429-62.66539\cdot e^{-0.00015874\cdot t}$	0.99363	101.02823	3.25898
MPAB	$Q_{t}=70.43298-64.81194\cdot e^{-0.0003305\cdot t}$	0.99087	207.23455	6.68499
	Higuchi model ($Q_{t}=K_{H}\cdot t^{1/2}+b_{H}$)			
	Equation	Adj. R-Square	Residual Sum of Squares	Reduced Chi-Sqr
PHBA	$Q_{t}=0.5015\cdot t^{1/2}-1.4566$	0.98813	188.83194	5.901
MPDB	$Q_{t}=0.6183\cdot t^{1/2}-1.1271$	0.9848	367.77507	11.49297
PABA	$Q_{t}=0.5086\cdot t^{1/2}-0.1951$	0.9886	186.48532	5.82767
MPAB	$Q_{t}=0.5936\cdot t^{1/2}+4.8542$	0.9385	1441.08361	45.03386
	Korsmeyer–Peppas model ($Q_{t}=K\cdot t^{n}$) $M_{t}/M_{\infty }<0.6$			
	Equation	Adj. R-Square	Residual Sum of Squares	Reduced Chi-Sqr
PHBA	$Q_{t}=0.3556\cdot t^{0.5381}$	0.99062	93.76934	3.34891
MPDB	$Q_{t}=0.2575\cdot t^{0.6062}$	0.9960	31.31753	1.3049
PABA	$Q_{t}=0.3593\cdot t^{0.5442}$	0.99655	31.14501	1.15352
MPAB	$Q_{t}=0.8642\cdot t^{0.4904}$	0.9895	80.37568	3.6534

**Table 2:** The release kinetics model fitting parameters of MPAB, EPAB, and BPAB drugs.

Drugs	$\mathrm{Zero}-\mathrm{Order}\;\mathrm{Release}\;\mathrm{Kinetics}\;(Q_{t}=K_{0}\cdot t+b_{0})$			
	Equation	Adj. R-Square	Residual Sum of Squares	Pearson’s r
MPAB	$Q_{t}=0.00412\cdot t+16.55115$	0.76907	5412.90171	0.88095
EPAB	$Q_{t}=0.00834\cdot t+52.90427$	0.49793	8485.70639	0.71973
BPAB	$Q_{t}=0.00848\cdot t+51.31287$	0.59024	6101.62499	0.77886
	First-order kinetics model ($Q_{t}=K_{1}\cdot e^{at}+b_{1}$)			
	Equation	Adj. R-Square	Residual Sum of Squares	Reduced Chi-Sqr
MPAB	$Q_{t}=70.43298-64.81194\cdot e^{-0.0003305\cdot t}$	0.99087	207.23455	6.68499
EPAB	$Q_{t}=92.19019-73.6779\cdot e^{-0.00418617\cdot t}$	0.97938	333.93063	14.51872
BPAB	$Q_{t}=90.68096-66.5947\cdot e^{-0.00334205\cdot t}$	0.96002	570.49626	24.80419
	Higuchi model ($Q_{t}=K_{H}\cdot t^{1/2}+b_{H}$)			
	Equation	Adj. R-Square	Residual Sum of Squares	Reduced Chi-Sqr
MPAB	$Q_{t}=0.5936\cdot t^{1/2}+4.8542$	0.9385	1441.08361	45.03386
EPAB	$Q_{t}=0.82613\cdot t^{1/2}+41.2667$	0.69225	5201.35896	216.723
BPAB	$Q_{t}=0.81728\cdot t^{1/2}+40.1615$	0.77359	3371.44973	140.47707
	Korsmeyer–Peppas model ($Q_{t}=K\cdot t^{n}$) $M_{t}/M_{\infty }<0.6$			
	Equation	Adj. R-Square	Residual Sum of Squares	Reduced Chi-Sqr
MPAB	$Q_{t}=0.8642\cdot t^{0.4904}$	0.9895	80.37568	3.6534
EPAB	$Q_{t}=6.2303\cdot t^{0.4333}$	0.9487	87.29838	10.912
BPAB	$Q_{t}=8.2661\cdot t^{0.3732}$	0.93087	93.49352	11.68669

**Table 3:** The release kinetics model fitting parameters of 2-MBA, 3-MBA, and 4-MBA drugs.

Drugs	$\mathrm{Zero}-\mathrm{Order}\;\mathrm{Release}\;\mathrm{Kinetics}\;(Q_{t}=K_{0}\cdot t+b_{0})$			
	Equation	Adj. R-Square	Residual Sum of Squares	Pearson’s r
2-MBA	$Q_{t}=0.00879\cdot t+59.55212$	0.39983	7510.16528	0.65263
3-MBA	$Q_{t}=0.00704\cdot t+69.74208$	0.3818	5172.79936	0.63928
4-MBA	$Q_{t}=0.00268\cdot t+20.23364$	0.69758	1667.87643	0.84122
	$\mathrm{First}-\mathrm{order}\;\mathrm{kinetics}\;\mathrm{model}\;(Q_{t}=K_{1}\cdot e^{at}+b_{1}$)			
	Equation	Adj. R-Square	Residual Sum of Squares	Reduced Chi-Sqr
2-MBA	$Q_{t}=92.50316-69.28947\cdot e^{-0.00577987\cdot t}$	0.98442	186.11992	8.86285
3-MBA	$Q_{t}=96.02382-56.6948\cdot e^{-0.00609705\cdot t}$	0.96805	255.16132	12.15054
4-MBA	$Q_{t}=43.28254-32.72384\cdot e^{-0.00126406\cdot t}$	0.94884	272.41353	9.72905
	$\mathrm{Higuchi}\;\mathrm{model}\;(Q_{t}=K_{H}\cdot t^{1/2}+b_{H}$)			
	Equation	Adj. R-Square	Residual Sum of Squares	Reduced Chi-Sqr
2-MBA	$Q_{t}=0.81544\cdot t^{1/2}+48.7023$	0.60773	4908.5863	223.11756
3-MBA	$Q_{t}=0.65637\cdot t^{1/2}+60.9671$	0.58745	3451.99712	156.908
4-MBA	$Q_{t}=0.34276\cdot t^{1/2}+14.1861$	0.88236	648.78545	22.37191
	$\mathrm{Korsmeyer}–\mathrm{Peppas}\;\mathrm{model}\;(Q_{t}=K\cdot t^{n}$ $)\;M_{t}/M_{\infty }<0.6$			
	Equation	Adj. R-Square	Residual Sum of Squares	Reduced Chi-Sqr
2-MBA	$Q_{t}=8.79065\cdot t^{0.40362}$	0.94053	61.20544	10.20091
3-MBA	$Q_{t}=14.4650\cdot t^{0.36644}$	0.97799	7.76072	2.58691
4-MBA	$Q_{t}=6.0925\cdot t^{0.22252}$	0.97279	150.07601	5.17503

## Data Availability

Data is unavailable due to privacy.
